# Two Ways to Frustrate a Desire

**DOI:** 10.1007/s10790-017-9586-9

**Published:** 2017-02-20

**Authors:** David Birks, Thomas Douglas

**Affiliations:** 1 University of Kiel, Arnold-Heller-Straße 3, Haus 28, 24105 Kiel, Germany; 2 The Oxford Research Centre in the Humanities (TORCH), University of Oxford, Woodstock Road, Oxford, OX2 6GG UK; 3 Uehiro Centre for Practical Ethics, Faculty of Philosophy, University of Oxford, St Ebbes Street, Oxford, OX1 1PT UK

Suppose that a person, call him Andrew, has a desire to drink a cup of tea. He boils the kettle, pours the hot water into the teacup, places the teacup on a side table, and sits down next to it to wait for the tea to brew. When Andrew looks away, and before he is able to satisfy his desire, Beata quickly pours Andrew’s tea into a nearby plant pot because she believes that caffeine is bad for Andrew’s health. Let us call this example *Tea Plant*.

Now imagine a different case. Andrew has the desire to drink a cup of tea, and then, rather than pouring away the tea, Beata sprays a chemical in the air that stops Andrew drinking that cup of tea: as Andrew begins to lift the cup to his lips, the spray extinguishes his desire to drink the tea. Andrew knows that Beata’s spray was the cause of this change. Let us call this example *Tea Spray*.^1^

In both *Tea Plant* and *Tea Spray*, Beata prevents Andrew’s desire to drink the cup of tea from being satisfied, and in both cases, this is plausibly pro tanto wrong—that is, Beata’s action in each case possesses some feature or features that render it wrong, absent defeaters.^2^ Nevertheless, we suspect that many would judge there to be a *moral* difference between these cases—they would judge that the intervention in Tea Spray is in some respect more pro tanto wrong than the intervention in Tea Plant. That is to say, there is some feature of Beata’s action in Tea Spray that renders it wrong, absent defeaters, and that is either not possessed by her action in Tea Plant, or would be more easily defeated in that case.

One way in which one might attempt to account for intuitive judgements about these cases would be to invoke the following principle:

The Internal-External Principle (IEP): It is in one respect more pro tanto wrong if B internally frustrates A’s desire that x—that is, prevents it from coming about that x by altering A’s desire that x—than if B externally frustrates A’s desire that x—that is, prevents it from coming about that x through altering A’s environment.

IEP seems able to account for the putative moral difference between Tea Plant and Tea Spray, for Tea Plant involves external desire frustration, while Tea Spray involves internal desire frustration. We think that the ability of IEP to capture intuitive differences between these cases gives us some reason to accept it, or at least, take it seriously as a moral hypothesis. However, perhaps some would have different intuitive responses to these cases; responses that do not support IEP. Still, we think there are at least two further reasons to take IEP seriously.

First, the idea contained in IEP is itself plausible. There is a sense in which internally frustrating a desire is more intrusive than externally frustrating it, and it would not be surprising if more intrusive means of frustrating desires were more morally problematic than less intrusive ones. Consider, by analogy, the morality of bodily interference. It would be widely accepted that nonconsensually interfering with a person’s body by physically constraining him—say, by locking him in a room or pinning him to the floor—is typically less problematic than nonconsensually internally interfering with his body by performing a surgical procedure. One plausible way in which we might account for this is by invoking the idea that the degree of physical invasiveness makes a moral difference.^3^ One could think of IEP as positing a parallel difference between different forms of interference with the mind. Altering a desire seems in one respect more mentally intrusive than merely externally frustrating it.^4^

Second, IEP seems potentially capable of accounting for some views that are widely held in public and academic debate regarding three types of intervention, which we will refer to collectively as the *Practical Cases*.

The first of these Practical Cases has been discussed in recent literature in criminal justice ethics. There has been some debate recently concerning whether and when criminal offenders might permissibly be offered, or forced to receive, mind-altering drugs designed to prevent them from committing future offences. On one side of the debate, the practice of imposing mind-altering drugs as a condition of early release or parole has been criticised on the ground that, when the only alternative is to remain incarcerated, an offender cannot give valid consent to receive a mind-altering drug, because the offer is coercive.^5^ The dominant response has been that, although offenders offered a choice between receiving mind-altering drugs and further incarceration face pressure to consent to receive the drugs, the pressure is not coercive, or does not undermine the offender’s autonomy. As such, in these circumstances, an offender could give valid consent to receive a mind-altering drug.^6^ Interestingly, a number of authors on both sides of this debate have assumed that it is more problematic to nonconsensually impose such drugs on an offender than it is to incarcerate an offender.^7^ IEP could potentially explain this.

IEP may also be able to account for a widely held view regarding ‘nudge’ techniques, the second of our Practical Cases. On one account, B nudges A when she makes it more likely that A performs some action, by triggering A’s automatic cognitive processes.^8^ One example of a nudge is placing pictures of diseased lungs on cigarette packets to create reactions of disgust in order to discourage the smoking of tobacco.^9^ Another example is placing food with high-fat content further from eye level than healthier food in a cafeteria in order to discourage people from eating the high-fat food.^10^ It is sometimes thought that nudging an individual so that she chooses not to do *x* is in some cases more morally problematic than simply prohibiting *x*.^11^ IEP may be able to justify such a view. Nudges may sometimes involve changing behaviour through altering desires, and according to IEP this is in one respect more problematic than externally frustrating these desires, for example, through legal prohibitions.^12^

Finally, IEP also seems capable of accounting for widely held moral qualms regarding hypnosis, brainwashing and subliminal advertising. These techniques are generally assumed to be highly morally problematic and certainly more problematic than external forms of desire frustration.^13^ Indeed, it has recently been argued that they should sometimes be criminalised on the basis that they constitute an objectionable form of mental interference.^14^ Once again, IEP may be able to accommodate these views, since hypnosis, brainwashing and subliminal advertising arguably all involve the internal frustration of desires, at least in their typical cases.

IEP thus gains initial plausibility from at least three sources. First, it can capture intuitions regarding certain imaginary cases such as those with which we began this paper. Second, it has some theoretical plausibility, independent of its plausible implications for these cases. Third, it is able to account for some widely held views in public and academic debate.

Nevertheless, the aim of this paper will be to subject IEP to critical scrutiny. We argue that IEP can be accepted only in a highly attenuated form—a form so attenuated that it lacks the most significant practical implications of the original version. In Section 1, we consider whether and how IEP might be modified to better accommodate a range of case-based intuitions. This leads us to replace IEP, as it is formulated above, with a more modest variant. In Section 2, we suggest that, though this modified variant of IEP handles a range of cases plausibly, the most promising explanation for its correctness appeals to a dubious thesis concerning the value of autonomous thought. We suggest that it may thus require further modification. Finally, in Section 3, we set out the implications of our modifications to IEP. We show that the resulting, heavily modified variant of IEP is unable to account for the wrongness of many common incidents of the three Practical Cases introduced above. This implies that those who hold that interferences involved in these Practical Cases are particularly morally problematic should either give up this view, or provide a different explanation for their wrongness.

Before we proceed to that task, however, we should make four clarifications regarding the content and scope of IEP. Recall that, according to IEP, it is in one respect more pro tanto wrong if B internally frustrates A’s desire that x than if B externally frustrates A’s desire that x. But what is it to frustrate a desire? We understand frustrating a desire to consist in intentionally bringing it about that the desire is not satisfied.^15^ This means that a desire can be frustrated without the person who has the desire being aware that the desire has been frustrated. It is thus important to distinguish frustrating a desire, and the feeling of frustration when a desire is not satisfied; the latter is not necessarily present when the former occurs. We assume also that a desire can be frustrated even if the desire is no longer present at the time the desired state of affairs would have occurred. For example, suppose that A desires at T_1_ that x occur at subsequent time T_2_, though at T_2_, B no longer desires x. Suppose further that B brings it about that x does not occur at T_2_. On our account, B has frustrated A’s T_1_ desire.^16^

Our second clarification concerns the concept of *altering* a desire. We earlier noted that one internally frustrates a desire when one frustrates the desire *by altering it.* We assume that there are three ways in which a desire could be altered:

1. the object of the desire is changed;
2. the strength of the desire is changed, either increased or decreased;^17^ and
3. the desire is extinguished, such that the person no longer has that desire.

We include within category (1) both cases in which the object of a desire is altered in such a way that a new desire is a created, so that the old desire is replaced with a new one, and cases (if there are any such) in which the object of the desire is altered but the desire survives the change—the alteration does not affect the identity of the desire.^18^ Similarly, we include within category (2) both changes in strength that involve replacing the initial desire with a new, weaker or stronger desire, and cases that instead involve altering the strength of a desire that survives the alteration. We remain silent on what determines whether a desire survives an alteration in content or strength.

Third, we should note that the categories of internal and external frustration of desires, as posited by IEP, are not exhaustive. There are other ways in which a desire could be frustrated. For example, B could frustrate A’s autonomous desire that x by:

(i) altering A’s other desires, for example, by strengthening competing desires.
(ii) introducing some entirely new desire in A, for example, a desire that not-x, without altering A’s existing desires.
(iii) altering A’s non-desire mental states, for example, beliefs.
(iv) altering A’s non-mental internal bodily states, for example, by inducing temporary paralysis.

None of these means of frustrating a desire would qualify as either internal or external desire frustration, as we have characterised those categories. They do not operate by altering A’s desire, but nor do they operate by altering A’s environment. In this paper, we will, as far as possible, remain silent on the moral status of frustrating an autonomous desire neither internally nor externally.

Finally, fourth, we exclude from the category of external desire frustration cases in which the environment is altered, and this prevents a person’s desire from being satisfied, but it does so via the means of altering that person’s mental or bodily states (including that person’s desires). Consider again Tea Spray, in which Beata exposes Andrew to a spray that extinguishes his desire to drink the cup of tea. Though there is a sense in which Beata here alters Andrew’s environment and this frustrates his desire, the environmental change exerts its effect by altering Andrew’s mental or bodily states (in this case, his desire to drink tea). We thus do not class it as an instance of external desire frustration. In Tea Plant, by contrast, the environmental manipulation does not frustrate Andrew’s desire by altering his mental or bodily states; it does so simply by making the tea unavailable.

## 1.

IEP, as formulated above, is susceptible to counterexamples. Consider the following case:

*Tea Reasons:* Andy has a desire to drink a cup of tea. He boils the kettle, pours the hot water into the teacup, places the teacup on a side table, and sits down next to it to wait for the tea to brew. Betty believes that caffeine is bad for Andy’s health. Before Andy is able to satisfy this desire to drink a cup of tea, and without asking Andy’s permission, Betty correctly tells Andy that drinking tea is bad for his health. Andy believes Betty’s claim, and takes it to give him conclusive reasons not to drink tea. As a result, his desire to drink the tea dissipates.

In Tea Reasons, Betty’s action clearly prevents the satisfaction of Andy’s desire to drink tea by altering that desire. Her action results in Andy’s desire to drink tea being extinguished. Thus, if IEP is correct, her intervention in this case is more pro tanto wrong in one respect than Beata’s intervention in Tea Plant. But this is implausible.^19^

We might wonder, however, whether it is possible to modify IEP so as to accommodate Tea Reasons. Consider:

*The Perception Variant:* It is in one respect more pro tanto wrong if B prevents the satisfaction of A’s desire that x by *non*-*perceptually* altering that desire than if B externally frustrates A’s desire that x.

We take it that B prevents the satisfaction of A’s desire that x *perceptually* just in case she alters the desire by changing A’s environment in a way that B perceives. Otherwise, she alters the desire non-perceptually. It is clear that, in Tea Reasons, Betty alters Andy’s desire perceptually: Andy’s desire is altered only because he *hears* certain utterances made by Betty. On the other hand, it is clear that, in Tea Spray, Beata alters Andrew’s desire non-perceptually. Andrew’s neural, and thus mental, states are altered via a biological process that does not result in his perceiving any environmental change.

The Perception Variant of the IEP seems capable of distinguishing Tea Reasons from Tea Spray, and may thus be capable of explaining why Tea Spray seems in one respect more pro tanto wrong than Tea Plant, but Tea Reasons does not. However, it fails to yield plausible verdicts regarding other cases that we might expect IEP to capture. We could modify Tea Spray so that perceptual processes are involved. Let us do this by imagining that rather than spraying a chemical in the air, Beata employs hypnosis to stop Andrew from drinking that cup of tea: she gently swings her pocket watch in front of his eyes, while talking softly to him. As Andrew begins to lift the cup to his lips, the hypnosis causes him, without his knowledge, to enter a hypnotic state, during which the hypnotist extinguishes his desire to drink the tea. To Andrew it appears that he has whimsically changed his mind. Let us call this case *Tea Hypnosis.*

Unlike Tea Spray, Tea Hypnosis plausibly involves perceptual alterations to Andrew’s desires: the hypnosis procedure operates through perceptual channels. The Perception Variant of the IEP thus cannot account for the intuitively plausible view that Tea Hypnosis is in one respect more pro tanto wrong than Tea Plant.^20^

Perhaps we can instead invoke:

*The Resistibility Variant:* It is in one respect more pro tanto wrong if B prevents the satisfaction of A’s desire that x by *irresistibly* altering *A’s* desire that x than if B externally frustrates A’s desire that x.

We take it that B irresistibly alters A’s desire that x if she alters it in such a way that it is not possible for A to prevent his desire that x from being altered.^21^ It might seem that in both Tea Spray and Tea Hypnosis, it is impossible for A to prevent his desire that x from being altered by B, whereas in Tea Reasons it is not. However, the difficulty with attempting to account for our intuitive reactions to Tea Reasons by invoking the Resistibility Variant is that it is not clear that the intervention in Tea Reasons really *is* resistible. It might be argued that when one is presented with a persuasive reason to do something one frequently has no choice but to form the desire to do that thing.^22^ Yet, even were it to turn out that the intervention in Tea Reasons is not resistible, we would, we think, find the intervention described in this case to be unproblematic.

A further possible explanation for the moral difference between *Tea Reasons* and *Tea Spray* (and *Tea Hypnosis*) would appeal to the role of the object’s autonomy of thought in these cases. It might be argued that *Tea Reasons* engages the autonomy of thought of its object in a way that *Tea Spray* and *Tea Hypnosis* do not. In accordance with this suggestion, we might modify IEP to:

*The Autonomy Variant:* It is in one respect more pro tanto wrong if B prevents the satisfaction of A’s desire that x by non-autonomously altering that desire than if B externally frustrates A’s desire that x.

B non-autonomously alters A’s desire when B alters A’s desire through means other than engaging A in autonomous thought. We believe that this variant yields plausible verdicts regarding the cases we have discussed. According to this variant, Tea Spray and Tea Hypnosis are in one respect more pro tanto wrong than Tea Plant and Tea Reasons. This is because Tea Spray and Tea Hypnosis operate by altering a desire in a way that does not involve the agent’s autonomous thought, whereas Tea Reasons operates by altering the agent’s desire *though engaging the agent in autonomous thought*, and Tea Plant does not operate by altering the agent’s desire at all.

The Autonomy Variant of IEP has the virtue of being somewhat theoretically plausible. More specifically, it is possible to situate the principle within a deep liberal tradition, including both Kant and Mill, which holds that autonomous thought is valuable in itself, and independently of the value of the conduct that it produces.^23^

To see this, consider why it should be thought pro tanto wrong to alter a person’s desires in a way that fails to engage the individual’s autonomous thought. The most obvious answer is that doing so fails to treat the individual as an autonomous agent. But note that *externally frustrating* an individual’s desire plausibly also fails to treat the individual as an autonomous agent. It involves closing off options in preference to engaging the individual in autonomous thought about which option to choose. Thus, on this answer, it is unclear why internally frustrating a desire without engaging a person’s autonomous thought should be considered in one respect more pro tanto wrong that externally frustrating it. On this answer, the Autonomy Variant of IEP looks unmotivated.

However, a better answer is available. According to this second answer, altering A’s desire by means other than engaging A in autonomous thought is problematic because it amounts to an *interference* with A’s autonomy of thought. When one alters a person’s desires through means that do not engage the individual in autonomous thought, and when one’s alteration of that person’s desires was not done on the basis of that person’s autonomous wishes, one plausibly interferes with this person’s autonomy of thought.^24^

If we accept that autonomous thought is valuable in itself, then it will be plausible to think that means of altering desires that involve interference with autonomy of thought are particularly problematic. It might, however, be objected that autonomy of action, the condition of acting autonomously, is also valuable, and externally frustrating a desire interferes with *that* form of valuable autonomy. However, even if this is so, it will remain plausible that frustrating a desire by altering it in a way that involves interference with autonomy of thought constitutes a more serious threat to valuable autonomy than externally frustrating the desire. This is because both kinds of desire-frustration interfere with autonomy of action, but the latter *also* interferes with autonomy of thought.

## 2.

We believe that the Autonomy Variant of IEP is the most plausible of the variants we have considered. However, in this Section, we offer a case for modifying the Autonomy Variant by challenging the most promising explanation for its correctness: that autonomy of thought is invariably valuable. In order to do this, we consider Joseph Raz’s argument concerning the conditional value of autonomy of action. We then apply Raz’s argument for the conditional value of autonomy of action to the value of autonomy of thought, which we suggest may also be conditional. If the value of autonomy of thought is recognised to be conditional, the most obvious explanation for the correctness of the Autonomy Variant becomes unavailable, and as a consequence, we tentatively propose a further modification of this variant.

Before we do this, we should clarify that we are not denying that there might be other possible explanations for the wrongness of interfering with autonomy of thought. We do, however, take an explanation appealing to the *value* of autonomy of thought to be the most promising candidate explanation. Thus, the problems faced by that explanation diminish the credence we should place in the Autonomy Variant of IEP.

According to Raz, “Autonomous life is valuable only if it is spent in the pursuit of acceptable and valuable projects and relationships”.^25^ Autonomy of action, that is, the condition of acting autonomously, can possess two kinds of value: the value of the acts it produces (action-value), and the value of the autonomy itself (autonomy-value).^26^ Suppose an autonomous agent exercises his autonomy by reading *War and Peace*. The action of reading this work may be valuable, either because the action of reading a great work of literature is an intrinsically valuable action, or because it has instrumental value, such as producing pleasure, knowledge, or something else of value. Thus, the agent’s autonomy with respect to this action has action-value. But it might also seem to possess autonomy-value. It might seem that it would have been in one way worse had the agent read *War and Peace* nonautonomously—say, at gunpoint.

On Raz’s view, however, the agent’s autonomy of action would not have had any autonomy-value had it been used to perform an *all things considered* disvaluable action.^27^ The presence of autonomy-value is conditional on the presence of action-value. If an agent autonomously performs a disvaluable action then the agent’s autonomy with respect to that action is not valuable in itself. For example, assume the action of torturing kittens is disvaluable and consider two possible states of affairs: in one, an agent autonomously tortures a kitten, in another, an agent nonautonomously tortures a kitten (say, at gunpoint). On Raz’s view, the presence of greater autonomy of action in the first state of affairs does not make that state of affairs in any respect better than the other. Let us summarise Raz’s view as the following:

*Conditional Action*: Autonomy of action lacks autonomy-value when employed to perform *all things considered* disvaluable actions.

Raz does not provide much of an argument in support of Conditional Action, and a comprehensive defence of this view is beyond the scope of the paper.^28^ Nevertheless, we will now quickly show why it may be a plausible position to hold.^29^ There is considerable intuitive force in the idea that it is not better in any respect when a person autonomously acts disvaluably than when a person performs the same disvaluable action nonautonomously. To illustrate this, consider the two following cases:

*Ted*: Ted hates Jill and plans to kill her if ever an opportunity arises to do so without being caught. One day such an opportunity does arise and Ted decides to take it; he kills Jill.

*Todd*: Todd hates Jill and plans to kill her if ever an opportunity arises to do so without being caught. This opportunity never arises. But one day he eats some wild berries containing a rare neurotoxin that temporarily deprives him of all impulse control while also producing in him a strong desire to kill Jill. He acts on this desire and kills her.

Let us assume that the action of killing Jill is *all things considered* disvaluable. Both Ted and Todd perform the same disvaluable action of killing Jill. However, Todd’s action of killing Jill is not autonomous because the desire that he acts on is determined by the neurotoxin. By contrast, Ted’s desire to kill Jill is an autonomous action. If we were to hold that autonomy of action is unconditionally valuable, we would have to say that there is something more valuable about the state of affairs described in the Ted case than about the state of affairs described in the Todd case. However, it is at the very least doubtful that this is so. This may provide (weak) support to Conditional Action.

### 2.1.

Suppose we tentatively accept Conditional Action. It might seem that we should then also accept that autonomy of thought – understood as the condition of forming and sustaining one’s desires autonomously – also possesses only conditional value. In particular, it might be thought that autonomy of thought lacks non-instrumental value when employed to form or sustain desires that are *all things considered* disvaluable.^30^ For example, the desire to gratuitously torture kittens is all-things-considered disvaluable, and consequently, the autonomous thought deployed to sustain the autonomous desire to gratuitously torture kittens is valueless. Let us refer to this as Conditional Thought.

Conditional Thought derives support from Conditional Action. If autonomy of action has autonomy-value only when deployed to perform valuable actions, it is plausible that a parallel claim will be true of autonomy of thought. This is because it is plausible that, if autonomy of action has only conditional value, this is because, quite generally, autonomy is valuable only if put to good use, and this more general view will support Conditional Thought.

Conditional Thought has significant implications for the Autonomy Variant. Recall that the plausibility of this variant was in part due to the fact that non-autonomously altering a desire amounts to an interference with autonomy of thought as well as with autonomy of action. It therefore interferes with autonomy to a greater extent than an external frustration of a desire, which only interferes with autonomy of action. This difference explained why an internal frustration of a desire is in one respect more pro tanto wrong than an external frustration of the same desire. However, on Conditional Thought, autonomy of thought is not valuable when used to sustain disvaluable desires, and we thus cannot invoke its value to explain why interfering with both autonomy of thought and autonomy of action is worse than interfering only with autonomy of action in such cases. This leaves us without an explanation for why nonautonomously altering A’s desire is more pro tanto wrong than externally frustrating the desire in cases where A’s autonomy of thought is being put to disvaluable use.

One response to this would be to seek an alternative explanation for why interfering with autonomy of action *and thought* is worse than interfering with autonomy of action alone—an explanation that does not invoke the value of autonomy of thought. One might, for instance, seek to show that, regardless of the value of autonomy of thought, we possess a right against interferences with that autonomy. We remain open to the possibility that this could be established, though we are not aware of any plausible explanation for why we should be thought to possess such a right.

Alternatively, we could seek to accommodate Conditional Thought by making a further modification to IEP:

*The Conditionalized Autonomy Variant:* If A’s desire that x is not all-things-considered disvaluable, it is in one respect more pro tanto wrong if B prevents the satisfaction of this desire by non-autonomously altering it than if B externally frustrates the desire.

In the following section, we will demonstrate the significant implications of moving to this variant of IEP. First, we should consider an objection against the argument of this section. We have argued that Conditional Action supports Conditional Thought, which in turn supports the view that it is not always more wrong to frustrate a desire by non-autonomously altering it than to externally frustrate it. However, one concern could be that we do not provide a theoretical justification for Conditional Action, but instead only rely on an example that illuminates its intuitive plausibility. But it might be objected that Conditional Action also has implications that are severely counterintuitive. For example, it might be thought that Conditional Action also shows directly that it is not at all wrong to *externally* frustrate a desire when one does so by preventing that desire from giving rise to a *disvaluable* action. In externally frustrating a desire in this way, one will plausibly be interfering with autonomy of action that possesses no autonomy-value according to Conditional Action. It might seem implausible, however, that it is in no way wrong to externally frustrate a desire in this way, even if the desire would give rise to a disvaluable action. It might be objected that this constitutes a reductio of our view. For this implication might seem far more implausible than the implications of rejecting Conditional Action. If intuitive plausibility is the basis to accept or reject a view of the value of autonomy, we should reject Conditional Action.

We accept that this is a reasonable concern about our view. Nevertheless, we think that this claim of intuitive implausibility can be, in part, defused. For it is not, we think, *clearly* in one way wrong to externally interfere with a desire by preventing it from giving rise to a disvaluable action. We can see this by considering certain other cases in which externally frustrating a desire is not in any way wrong. Suppose C sees two strangers in the park, and then has a desire that stranger A declares her love for stranger B. Stranger A elects not to do so, thus externally frustrating C’s desire. It is not clear that A has done anything even *pro tanto* wrong. For it is none of C’s business what intimate things one stranger says to another. In this case, C has a desire about something that does not properly fall within the scope of C’s autonomy, and for this reason, one could not appeal to C's autonomy to show that it is in no way wrong for A to externally frustrate C’s desire. But the same thoughts could plausibly be invoked in relation to cases where we frustrate autonomous desires by preventing them from giving rise to disvaluable actions: it is not clear that performing disvaluable actions falls within the proper scope of one’s autonomy.

Moreover, even if it is always in at least one way wrong to externally frustrate a desire, it may be possible to endorse Conditional Action. This is because externally frustrating a desire need not be wrong *because* it constitutes an interference with valuable autonomy of action, rather, there could be other reasons why it is wrong. For example, one could appeal to political liberalism and argue that, due to reasonable disagreement, it is impermissible for the state to interfere externally (or indeed internally) with actions that it judges to be disvaluable, as long as those actions are reasonable.^31^ Crucially, the impermissibility of this interference is grounded on concerns that are arguably distinct from autonomy, namely the requirement to respect the equal moral status of each citizen or to remain neutral been alternative conceptions of the good.^32^ This could account for the view that it is wrong in at least one respect to frustrate a disvaluable desire externally, at least in cases where doing so involves interfering with reasonable actions. But it does not undermine Conditional Action, since it does not invoke the unconditional value of autonomy of action. It thus does not undermine our argument for modifying the Autonomy Variant to the Conditionalized Autonomy Variant.

A related objection has been raised by an anonymous reviewer, namely, that Conditional Thought could permit reprehensible mind control. If autonomy of thought lacks non-instrumental value when employed to form or sustain desires that are *all things considered* disvaluable, then it might be objected that it would permissible to non-autonomously intervene to alter these non-instrumentally disvaluable thoughts. This objection parallels the previous one, except that it appeals to the implausibility of it being permissible to interfere with disvaluable *desires*, rather than to the implausibility of it being permissible to interfere with disvaluable actions.

Again, we accept that this is a legitimate concern. In particular, there are difficulties surrounding the correct identification of non-instrumentally disvaluable autonomy of thought, and as such it is possible that the intervener would make mistaken judgements and interfere with valuable autonomy of thought. Moreover, the method of interfering with non-instrumentally disvaluable thoughts could result in interferences with thoughts that are not non-instrumentally disvaluable. For example, when administering testosterone-reducing drugs on sex offenders this might not only extinguish disvaluable desires, but could also extinguish non-disvaluable desires to have permissible sexual relationships.^33^ We think that once we set aside all of the considerable and significant instrumental reasons against non-autonomously interfering with (even disvaluable) desires, it is not obviously wrong to interfere such desires.^34^ But even if, setting aside instrumental reasons, it *would* be wrong to non-autonomously interfere with an individual’s disvaluable desires, it would not follow that autonomy of thought is unconditionally valuable, for such interferences could be wrong for reasons other than that they interfere with value. For example, we could again appeal to the liberal view that interfering with a person’s desires is pro tanto wrong insofar as it involves a violation of equal respect or of liberal neutrality. Importantly, it would not obviously follow from such an appeal that interfering with desires and actions is in any respect *more* pro tanto wrong than interfering with actions alone, since it is not obvious that interfering with a person’s desires and actions constitutes a graver violation of equal respect or liberal neutrality than interfering with actions alone.

## 3.

Recall that, in Section 1, we stipulated that the initial version of IEP could potentially account for commonly held views concerning the wrongness of three controversial kinds of interferences, referred to as the Practical Cases. Namely, we suggested that IEP may be able to account for the views that (i) imposing mind-altering drugs on criminal offenders is in one respect more pro tanto wrong than incarcerating them, (ii) nudging people away from certain behaviours is more pro tanto wrong than prohibiting those behaviours, and (iii) hypnosis, brainwashing and subliminal advertising are more pro tanto wrong that comparable forms of external desire frustration. In each case, some forms of internal desire frustration are considered to be more pro tanto wrong in one respect than comparable forms of external desire frustration. For the sake of brevity, henceforth we refer to an interference that is more pro tanto wrong in one respect than external desire frustration as having *special* wrongness.

While the initial variant of IEP could account for the special wrongness of the Practical Cases, in this section we demonstrate that once we have revised IEP, the number of Practical Cases involving special wrongness is significantly reduced. We show that the Conditionalized Autonomy Variant does not account for the special wrongness of many of the most common incidents of the Practical Cases. This is unlikely to be satisfactory for those who hold that the interferences in the Practical Cases have special wrongness. As a result, those who hold that there is a special wrongness in the Practical Cases should either provide a different explanation for the special wrongness in the Practical Cases, or give up the view that the internal interference in the Practical Cases are more pro tanto in one respect than an external interference.

Let us consider the implications of the Conditionalized Autonomy Variant for the wrongness of the Practical Cases. We suggested in Section 2 that it is not, by reason of the value of autonomy of thought, invariably more pro tanto wrong in one respect to frustrate a disvaluable desire by non-autonomously altering it than to externally frustrate a disvaluable desire. This led us to tentatively adopt the Conditionalized Autonomy Variant of IEP, which holds only that frustrating a *non*-*disvaluable* desire by non-autonomously altering it is more pro tanto wrong in one respect than externally frustrating it. But the three Practical Cases can often involve interfering with *dis*valuable desires and thus fall outside of the scope of this variant of IEP. In some instances of criminal offenders being forced to take mind-altering drugs, the interference alters the agent’s desire to commit a crime. Although there is no necessary connection between the value of desires and the lawfulness of the actions it motivates, it is plausible that many desires to commit crimes are all-things-considered disvaluable. The Conditionalized Autonomy Variant cannot explain why frustrating these desires by non-autonomously altering them is in one respect more pro tanto wrong than externally frustrating them.^35^

Let us now turn to the implications of the Conditionalized Autonomy Variant for the wrongness of deploying nudge techniques. This variant entails that the non-autonomous alteration of desires has special wrongness if the altered desires are not all-things-considered disvaluable. But many nudge techniques that have been used or advocated plausibly involve altering desires that *are* all-things-considered disvaluable.^36^

Consider nudges designed to prevent eating fatty food and smoking tobacco. It is plausible to assume that these are typically both pleasurable activities, and that the pleasure derived from these activities is valuable. However, both activities can result in significant harm to the health of the agent, and it is plausible that the disvalue of this harm frequently exceeds the value of the short-term pleasure produced by the activities.^37^ More generally, many proposed nudge techniques seek to alter desires that are plausibly *all things considered* disvaluable, and this should not surprising, since negative evaluations of these desires presumably provide much of the motivation for introducing these nudges.^38^

Finally, consider the third of the Practical Cases, namely, hypnosis, brainwashing, and subliminal advertising. While the other two Practical Cases commonly involve interventions with a person’s disvaluable desires, these cases are more frequently associated with interference with a person’s *valuable* desires for the purposes of producing disvaluable desires or actions. For instance, examples of subliminal advertising include moving people to buy things that they do not need, or brainwash and hypnotise people to act in ways that are disvaluable.^39^ In these cases, where the hypnosis, brainwashing, and subliminal advertising non-autonomously alters a person’s valuable desire, then the Conditionalized Autonomy Variant can account for the special wrongness of these cases. However there could, of course, be cases in which interventions of this sort are used to non-autonomously alter disvaluable desires—consider a case in which B hypnotises A not to act in accordance with A’s autonomous desire to murder her neighbour—and in these cases the Conditionalized Autonomy Variant will not imply that the intervention is in one respect pro tanto wrong than an external interference with the same desire.

## Conclusion

In this paper, we considered several variants of IEP, and showed that each was susceptible to counterexamples. The first variant of IEP could not offer a plausible verdict on Tea Reasons, which involved a morally unproblematic means of desire frustration. A further variant, the Perception Variant, gave a plausible verdict on Tea Reasons, but could not account for the wrongness Tea Hypnosis. We then considered the Resistibility Variant of IEP, but this was also shown to inadequately capture the wrongness of these cases.

We next considered the Autonomy Variant, which circumvented the difficulties faced by the other IEP variants, while also being theoretically plausible. While an external interference frustrates autonomy of action, an internal interference also interferes with autonomy of thought. We suggested that this constitutes a more serious interference with autonomy, and so can account for the view that internal desire frustration is in one respect more pro tanto wrong than external desire frustration. Nevertheless, we tentatively proposed that the Autonomy Variant might require further modification because it may be grounded on a dubious view of the value of autonomous thought. We considered Joseph Raz’s argument concerning the conditional value of autonomy of action and applied Raz’s argument to the value of autonomous thought, which we supposed is also conditional. Once this conditional view of the value of autonomous thought is adopted, we showed that the Autonomy Variant loses its most obvious explanatory support, and as a consequence, we tentatively proposed a further variant of IEP: the Conditionalized Autonomy Variant.

In the final section of the paper, we showed that our weakening of IEP has significant implications for the wrongness of the interferences in the Practical Cases. We showed that on Conditionalized Autonomy Variant, many instances of the Practical Cases do not have special wrongness. This is because a number of common instances of the Practical Cases involve internal non-autonomous alteration of desires that are all-things-considered disvaluable. Those who hold that interferences in these Practical Cases are particularly morally problematic even when the altered desires are all-things-considered disvaluable should either provide a different explanation for the wrongness of these interferences, or give up the view that these interferences are in one respect more pro tanto wrong than the external frustration of the same desires.

## Footnotes

1 To clarify, the spray has no other effect apart from extinguishing this desire. Also, we assume for the sake of consistency across cases that the spray only stops Andrew from drinking the cup of tea at that time. It does not prevent Andrew from drinking cups of tea in the future.

2 Unless otherwise stated, when we claim that an action is wrong we mean pro tanto wrong, that is, wrong absent defeaters. We take it that the pro tanto wrongness of an action is a matter of degree such that, the greater an action’s pro tanto wrongness, the stronger the defeating consideration would need to be for the action to be morally permissible—i.e., not morally wrong—*all things considered*.

3 As is often the case, the law follows common sense morality here, treating more physically invasive forms of interference as more serious (legal) wrongs. For example, in England and Wales, non-consensual bodily interference that involves physical contact constitutes the tort of battery, which is actionable *per se*, but battery is not available in respect of interventions that do not involve physical contact, since touching is an element of the tort; here negligence is the only potential source of liability (Chatterton v Gerson [1981] QB 432), and a patient could bring a successful claim in negligence only if she could establish injury and causation. Moreover, within the class of interferences that involve physical contact, those that are more physically invasive are often treated as more serious wrongs. For example, in England and Wales, any non-consensual bodily interference constitutes the tort battery and therefore incurs potential civil liability, but bodily interference that breaks the skin would normally also constitute ‘actual bodily harm’ (ABH) or more serious injury, which could be a criminal offence contrary the Offences Against the Person Act 1861, ss 18, 20 or 47 (depending on severity of injury and intent). We thank Lisa Forsberg for her helpful comments on this point.

4 We take it that where an intervention falls on the internal-external divide is one determinant of its intrusiveness, but that there may also be other factors. For example, within the category of internal interferences there may be differences in intrusiveness due to, say, the centrality of the altered desire to the person’s personality.

5 K.A. Vanderzyl. Castration as an Alternative to Incarceration: An Impotent Approach to the Punishment of Sex Offenders. *Northern Illinois University Law Review* 1994; 15: 107–140, W. Green. Depo-Provera, Castration, and the Probation of Rape Offenders. *University of Dayton Law Review* 1986; 12: 1–26.

6 C.S. Rosati. A Study of Internal Punishment. *Wisconsin Law Review* 1994; 1994: 123–170, J. Ryberg. Punishment, Pharmacological Treatment, and Early Release. *International Journal of Applied Philosophy* 2012; 26: 231–244. A. Wertheimer & F. Miller, G. There Are (STILL) No Coercive Offers. *Journal of Medical Ethics* 2013, J. Ryberg & T. Petersen. Neurotechnological Behavioural Treatment of Criminal Offenders—A Comment on Bomann-Larsen. *Neuroethics* 2013; 6: 79–83.

7 See, for example, Scott, C. L, and T. Holmberg. “Castration of Sex Offenders: Prisoners’ Rights Versus Public Safety.” Journal of the American Academy of Psychiatry and the Law 31, no. 4 (2003): 502–509; Vanderzyl, Kari A. “Castration as an Alternative to Incarceration: An Impotent Approach to the Punishment of Sex Offenders.” Northern Illinois University Law Review 15 (1995 1994): 107–140; Green, W. “Depo-Provera, Castration, and the Probation of Rape Offenders: Statutory and Constitutional Issues.” U. Dayton L. Rev. 12 (1986): 1–26; Bomann-Larsen, Lene. “Voluntary Rehabilitation? On Neurotechnological Behavioural Treatment, Valid Consent and (In)appropriate Offers.” *Neuroethics* 6, no. 1 (April 1, 2013): 65–77. These authors assume that anti-libidinal medications should be provided to sex offenders only with their valid consent, though they do not object to nonconsensual incarceration.

8 This is an adapted definition of nudging from Y. Saghai. Salvaging the concept of nudge. *Journal of Medical Ethics* 2013, R.H. Thaler & C.R. Sunstein. 2008. *Nudge: improving Decisions about Health, Wealth, and Happiness*. London: Yale University Press.

9 C.R. Sunstein. 2013. *Simpler: The Future of Government*. New York: Simon & Schuster. p. 133. Importantly, there is a difference between displaying images to create disgust and providing statistics with the aim of informing people about the risks of smoking. The latter is not a nudge because it does not depend on a trigger of a person’s automatic cognitive processes.

10 Thaler & Sunstein. 2008. Nudge: improving decisions about health, wealth, and happiness. p. 1.

11 For evidence that supports that covert influences on behaviour are considered less objectionable than overt influences see: G. Felsen, et al. Decisional enhancement and autonomy: public attitudes towards overt and covert nudges. *Judgment and Decision Making* 2013; 8: 202–213.

12 T. Goodwin. Why We Should Reject ‘Nudge’. *Politics* 2012; 32: 85–92.

13 See for instance: R. Crisp. Persuasive Advertising, Autonomy, and the Creation of Desire. *Journal of Business Ethics* 1987; 6: 413–418.

14 J.C. Bublitz & R. Merkel. Crimes Against Minds: On Mental Manipulations, Harms and a Human Right to Mental Self-Determination. *Criminal Law and Philosophy* 2012.

15 We also assume that in all of the cases discussed throughout the paper, the interfering agent acts with the intention that the other agent’s desire is not satisfied. Although some may hold that the wrongness of the interference would change if the interfering agent did not intend to frustrate the desire, with this assumption, we set aside whether this intention of the interfering agent affects the permissibility of his actions. For discussion of this debate, see: Victor Tadros. 2012. *The Ends of Harm: The Moral Foundations of Criminal Law*. Oxford: Oxford University Press. Chapter 7.

16 It could be objected that a T_1_ desire that x occur at T_2_ is frustrated by an interference only if the agent would have continued to have this desire at T_2_ had the interference not taken place. We disagree, and think that it is plausible to think that a T_1_ desire that x occur at T_2_ can be frustrated even if the desire would no longer have existed at T_2_. We think this can be seen by considering cases of posthumous desire frustration. Suppose A desires that after her death her work will be respected. Suppose that her work is not respected after her death. It seems to us that A's desire is frustrated, even though the desire would not exist after her death.

17 It might be unclear how an increase in strength of a desire could result in the frustration of that desire, but it is possible to imagine cases in which this occurs. Suppose that Ruth desires to be friends with Ollie, but this desire is so strong that her behavior becomes obsequious—she laughs too loudly at his jokes, offers too much help—to the extent that her behavior becomes annoying to Ollie. In this case, the strength of her desire to be friends with Ollie makes it less likely that her desire is satisfied, than if it were less strong.

18 Suppose that Jack has a desire to eat ice cream. He then sees a passerby eating chocolate ice cream. This reminds him how much he likes chocolate ice cream, which results in his desire to eat ice cream being transformed into a desire to eat *chocolate* ice cream specifically. We take it to be somewhat plausible (though certainly not obvious) that Jack’s earlier desire to eat ice cream and later desire to eat chocolate ice cream are different phases of the same desire, rather than numerically distinct desires.

19 It has, however, been suggested that rational persuasion is not without its problems. See: R. Nozick. 1981. *Philosophical Explanations*. Cambridge, Massachusetts. pp. 4–8. G. Tsai. Rational Persuasion as Paternalism. *Philosophy and Public Affairs* 2014; 42: 78–112.

20 Tea Hypnosis might seem in one respect less morally problematic than Tea Spray. Moreover, the Perception Variant of IEP might account for this difference. However, Tea Hypnosis still seems more pro tanto wrong in one respect than Tea Plant, and the Perception Variant cannot account for *that* difference. Moreover, we are inclined to think that this is the greater difference: it seems to us that Tea Hypnosis is, morally speaking, much closer to Tea Spray than to Tea Plant.

21 There are a number of important issues here concerning the notion of possibility that we are unable address in this paper. For instance we can follow Parfit and draw the distinction between ‘Deep’ and ‘Technical’ impossibilities. Deep impossibilities contravene laws of nature, whereas technical impossibilities are presently impossible but could be possible in the future with the sufficient development of technology and science. See: D. Parfit. 1984. *Reasons and Persons*. Oxford: Oxford University Press. p. 219.

22 One related view is that a person cannot choose one’s beliefs, see: J. Raz. 1999. *Engaging Reason*. Oxford: Oxford University Press. Chapter One.

23 See: J.S. Mill. 1985. *On Liberty*. London: Penguin Classics. I. Kant. 2005. Critique of Practical Reason. M. Gregor, ed. Cambridge: Cambridge University Press. p. 12. We are not denying that there are important differences between Kant and Mill with regards to the value of autonomy.

24 This could be explained by an appeal to a historical account of autonomy, according to which a desire is autonomous only if produced by that individual’s autonomous thought. See for instance, J. Christman. Autonomy and Personal History. *Canadian Journal of Philosophy* 1991; 21: 1–24. When one alters a desire in a way that doesn’t engage (and was not the product of) that person’s autonomy of thought, one is thus effectively inserting a non-autonomous desire.

25 We understand autonomous life here to be referring to autonomy of action. As will be clear in the following discussion, the projects and relationships can be either intrinsically or instrumentally valuable. In places it seems that Raz also holds that that autonomously acting disvaluably *diminishes* a person’s wellbeing. For present purposes we will simply assume that it merely is not good in any way, and remain silent on whether it is an additional bad.

26 We assume that the value of the action is independent of the autonomousness of the action.

27 We henceforth frequently omit ‘*all things considered*’. ‘Disvaluable’ should be taken to mean ‘*all things considered* disvaluable’ unless otherwise specified.

28 Jeremy Waldron criticises Raz’s argument on the basis that it seems to equate the value of autonomy with the moral praiseworthiness of the person. J. Waldron. Autonomy and Perfectionism in Raz's Morality of Freedom. *Southern Californian Law Review* 1989; 62: 1097–1152. p.1100.

29 Raz claims that Conditional Action refers to both prudential and moral disvalue. Raz holds there is no meaningful difference between moral and prudential value, as they are both grounded from the same source. See: J. Raz. 1986. *The Morality of Freedom*. Oxford: Clarendon Press. pp. 313–320. We are open to the possibility that Conditional Action should be restricted to a more narrowly defined form of value, but for the purposes of this paper it makes little difference in terms of the implications for many of the Practical Cases.

30 Again, we henceforth frequently omit the ‘*all things considered*’.

31 See Raz. 1999. Engaging Reason. For variations of this view see: J. Rawls. 1999. *A Theory of Justice*. Revised edn. Cambridge, MA: The Belknap Press of Harvard University Press. J. Rawls. 2005. *Political Liberalism*. New York: Columbia University Press.

32 For example, Jonathan Quong rejects the view that autonomy can ground the wrongness of paternalistic interference, and instead argues that its wrongness can instead be grounded on the moral status of the intervenee. See: J. Quong. 2011. *Liberalism Without Perfection*. Oxford: Oxford University Press. pp. 96–101.

33 P. Briken & M.P. Kafka. Pharmacological treatments for paraphilic patients and sexual offenders. *Current Opinion in Psychiatry* 2007; 20: 609–613.

34 For a defence of grounding the wrongness of interference on contingent factors see: Raz. 1986. The Morality of Freedom. As a response, Quong considers the possibility of controlling a person’s preferences with a brain chip. He argues that even if the chip had the effect of interfering with only what we call nondisvaluable desires, it would be still intuitively troubling. Nevertheless, Quong also accepts that this is not decisive. Quong. 2011. Liberalism Without Perfection. pp. 54–56.

35 Note that our view can explain why even if mind-altering drugs deployed to change the sexuality of homosexuals were effective, the internal frustration of homosexual desires through chemical castration would be more wrong than externally frustrating them, because homosexual desires are normally all-things-considered valuable.

36 To clarify, here we mean that the desire is disvaluable *all things considered*, taking into account disvalue for the individual, disvalue for others, and disvalue from an impersonal point of view. It might be the case that the desire to smoke is disvaluable for the smoker, but valuable in some respect for others, such as the person who sells cigarettes. We are unable to address this issue adequately here, but it seems plausible to hold that in these typical cases, the fact that the desire is valuable for others will not be sufficient to result in an otherwise disvaluable desire being *all things considered* valuable. We are grateful for an anonymous reviewer for raising this point.

37 This could be because the health-effects themselves diminish future pleasure, or because they are disvaluable in some other way.

38 If pace Raz, one holds that Conditional Action only applies to moral value, and not *all things considered* value, then nudge techniques that interfere with prudentially disvaluable but morally positive or neutral desires might still have special wrongness according to the Conditionalized Autonomy Variant.

39 For example, in the film The Naked Gun, Jane is hypnotized to murder the Queen.

